# Loss of Bcl-G, a Bcl-2 family member, augments the development of inflammation-associated colorectal cancer

**DOI:** 10.1038/s41418-019-0383-9

**Published:** 2019-07-11

**Authors:** Paul M. Nguyen, Laura F. Dagley, Adele Preaudet, Nga Lam, Maybelline Giam, Ka Yee Fung, Kaheina Aizel, Gemma van Duijneveldt, Chin Wee Tan, Yumiko Hirokawa, Hon Yan K. Yip, Christopher G. Love, Ashleigh R. Poh, Akshay D’ Cruz, Charlotte Burstroem, Rebecca Feltham, Suad M. Abdirahman, Kristy Meiselbach, Ronnie Ren Jie Low, Michelle Palmieri, Matthias Ernst, Andrew I. Webb, Tony Burgess, Oliver M. Sieber, Philippe Bouillet, Tracy L. Putoczki

**Affiliations:** 1 The Walter and Eliza Hall Institute of Medical Research, Parkville, VIC 3052 Australia; 2 The Department of Medical Biology, The University of Melbourne, Melbourne, VIC 3050 Australia; 3 Now located at Monash Biomedicine Discovery Institute and Department of Biochemistry and Molecular Biology, Monash University, Clayton, VIC 3800 Australia; 4 Research Division, Peter MacCallum Cancer Centre, 305 Grattan St, Melbourne, Australia; 5 Olivia Newton-John Cancer Research Institute, and School of Cancer Medicine, La Trobe University, Heidelberg, VIC 3084 Australia; 6 Department of Surgery, The University of Melbourne, Melbourne, VIC 3052 Australia; 7 Department of Biochemistry & Molecular Biology, Monash University, Clayton, VIC 3800 Australia

**Keywords:** Cancer models, Tumour-suppressor proteins

## Abstract

Gastrointestinal epithelial cells provide a selective barrier that segregates the host immune system from luminal microorganisms, thereby contributing directly to the regulation of homeostasis. We have shown that from early embryonic development Bcl-G, a Bcl-2 protein family member with unknown function, was highly expressed in gastrointestinal epithelial cells. While Bcl-G was dispensable for normal growth and development in mice, the loss of Bcl-G resulted in accelerated progression of colitis-associated cancer. A label-free quantitative proteomics approach revealed that Bcl-G may contribute to the stability of a mucin network, which when disrupted, is linked to colon tumorigenesis. Consistent with this, we observed a significant reduction in Bcl-G expression in human colorectal tumors. Our study identifies an unappreciated role for Bcl-G in colon cancer.

## Introduction

Defects in the single layer of epithelial cells that line the gastrointestinal (GI) tract are implicated in the pathogenesis of inflammatory bowel disease (IBD) and colorectal cancer (CRC). In IBD patients, epithelial apoptosis is greatly enhanced at acute inflammatory sites and is associated with mucosal ulceration [1]. In parallel, persistent oxidative damage, as a result of the chronic inflammatory environment created by mucosal ulcers, can trigger mutagenic events in GI epithelial cells [2]. As a result, IBD patients have an increased risk of developing CRC [3].

BCL-G, also known as BCL2L14, is a poorly-studied member of the Bcl-2 family of proteins. The Bcl2 family is conserved throughout mammalian species, and each member harbors up to four Bcl-2 homology (BH) domains. Multi-domain pro-apoptotic proteins Bak and Bax trigger permeabilization of the mitochondrial membrane and the initiation of the intrinsic cell-death cascade [4]. Bcl-2, Bcl-X_L_, Bcl-w, Mcl-1, and A1 form the pro-survival subgroup, and each contain four BH domains (BH1 to BH4). These proteins bind to Bak and Bax, thereby inhibiting their function [5]. Pro-apoptotic BH3-only proteins Bad, Bid, Bik, Bim, Bmf, Hrk, Noxa, and Puma only contain the BH3 domain, which allows them to bind to and inhibit the pro-survival proteins, with some of them also described as direct activators of Bax and Bak [4, 6]. It is well established that the fate of a cell depends on the balance between the pro-survival and pro-apoptotic activities within this family, and their control of the integrity of mitochondria. The release of mitochondrial cytochrome c into the cytoplasm is the defining event that triggers the apoptotic cascade leading to cell death.

In humans (h) there are two BCL-G isoforms, hBCL-Gs and hBCL-G_L_, generated by alternative splicing [7]. hBCL-G_L_ contains BH2 and BH3 domains, with no other members of the Bcl-2 family containing only these two domains [7]. hBCL-G_S_ only harbors the BH3 domain, which is recognized as the critical death domain, leading to the suggestion that it is a member of the pro-apoptotic Bcl-2 family subgroup [7]. Overexpression of hBCL-G_S_ has been linked to cell death due to the capacity of its BH3 domain to neutralize the function of BCL-X_L_, this effect was less pronounced with hBCL-G_L_ [7]. In contrast, the BH2 domain from hBCL-G_L_ has been suggested to inhibit its interaction with BCL-X_L_ [7]. In mice (m), only one form of Bcl-G exists, which is 68% identical to hBCL-G_L_ and is expressed in similar tissues [7, 8]. Interestingly, while mBcl-G is highly expressed in the epithelial cells lining the GI tract, its role in this cell population is not known [8]. mBcl-G does not interact with the pro-survival members of the Bcl-2 family through its BH3 domain, suggesting that it is not directly involved in the intrinsic cell-death pathway [7, 9]. Similarly, hBcl-G_L_ did not induce cytoprotective properties when directly compared to Bcl-2 and Bcl-X_L_ [7]. The evolutionary significance of the potentially divergent function of the two hBCL-G isoforms remains unclear.

*BCL-G* is located on chromosome 12p12, which is lost in prostate cancers, ovarian cancers, and childhood acute lymphocytic leukemias [10–13]. Based on these observations, it has been assumed that BCL-G has a tumor suppressive function, although this has not formally been shown. We find that in mice devoid of *Bcl-g* the progression of colitis-associated cancer (CAC) is enhanced. Interestingly, we find that Bcl-G may contribute to regulation of the mucin network, which is essential to epithelial barrier function and, when disrupted, is known to contribute to CRC progression. The tumor suppressive phenotype associated with *Bcl-g* is consistent with the observation that *BCL-G* expression is reduced in late stage human CRC tumors.

## Results

### Loss of Bcl-G augments colitis-associated cancer

**Fig. 1:**
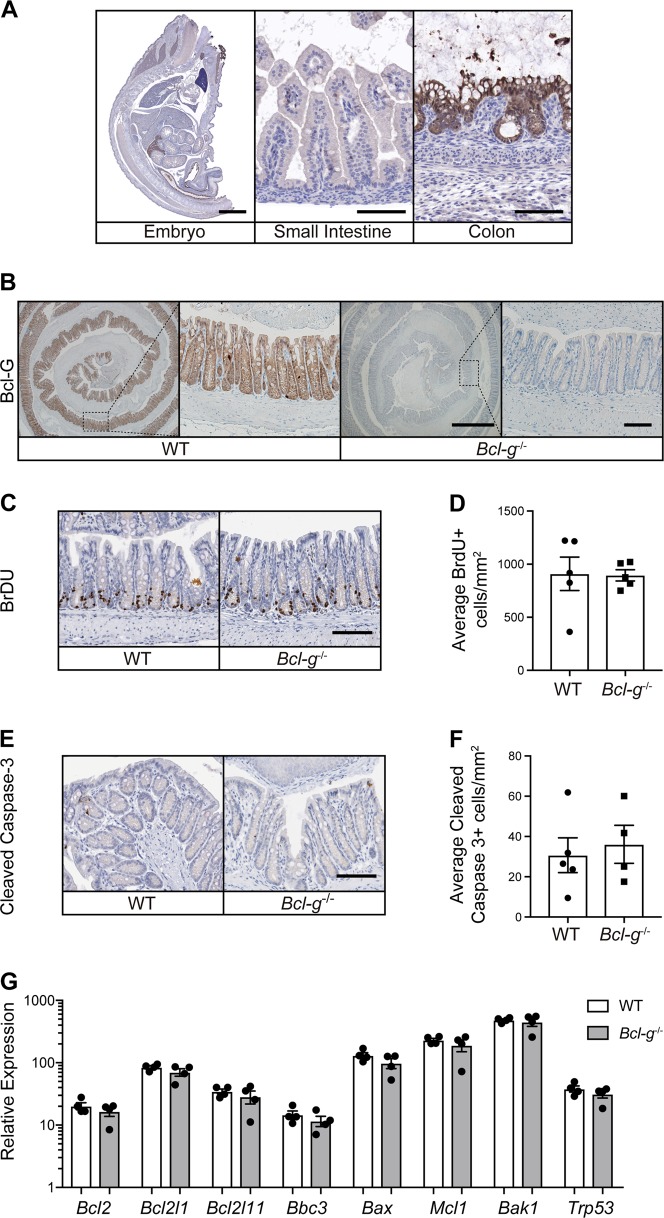
Gastrointestinal homeostasis is unperturbed in *Bcl-g*^−/−^ mice. **a** Representative Bcl-G immunohistochemistry of an E18.5 C57BL/6 mouse embryo (left; Scale bar: 2 mm). Representative images of the gastrointestinal epithelial cells of the small intestine (middle) and colon (right) are shown. Scale bar: 100 µm. **b** Representative immunohistochemistry for Bcl-G in the distal colon of 8-week-old WT and *Bcl-g*^−/−^ mice. Scale bar: 0.5 mm, inset 50 µm. **c**, **d** Representative immunohistochemistry for BrdU in the distal colon of 8-week-old WT and *Bcl-g*^−/−^ mice (**c**). Scale bar: 100 µm. Quantification of BrdU positive (+) staining for individual mice is shown (**d**). **e**, **f** Representative immunohistochemistry for cleaved Caspase-3 in the colon of 8-week-old WT and *Bcl-g*^−/−^ mice (**e**). Scale bar: 100 µm. Quantification of Caspase-3-positive (+) staining for individual mice is shown (**f**). **g** mRNA expression of the indicated genes from distal colonic tissue of individual age- and gender- matched 8-week-old WT and *Bcl-g*^-/-^ mice. *N* = 4 mice per genotype. Data presented are mean ± SEM

Bcl-G is expressed by colonic epithelial cells as early as embryonic day 18.5 (E18.5) (Fig. 1a) and remains highly expressed by epithelial cells lining the colonic crypts of adult mice (Fig. 1b). Surprisingly, naïve *Bcl-g*^*−/−*^ mice had normal colonic crypt architecture (Supplemental Fig. 1a), with no significant differences in the individual crypt lengths when compared to littermate WT mice (Supplemental Fig. 1b). This suggests that Bcl-G is not required for the normal proliferative turnover of colonic epithelial cells. This observation was validated by a lack of change in the number of epithelial cells entering S-phase, indicated by BrdU incorporation (Fig. 1c, d). We also observed no change in the number of cleaved Caspase-3-positive cells in the differentiated surface epithelium or within the proliferative zone of crypts (Fig. 1e, f), indicating that loss of Bcl-G does not affect normal apoptosis. Moreover, there was no change in the expression of other Bcl-2 family members within the colon of *Bcl-g*^−/−^ mice, making it unlikely that the function of Bcl-G in adult colonic crypts was masked by compensatory expression of other members of the Bcl-2 protein family (Fig. 1g).

**Fig. 2:**
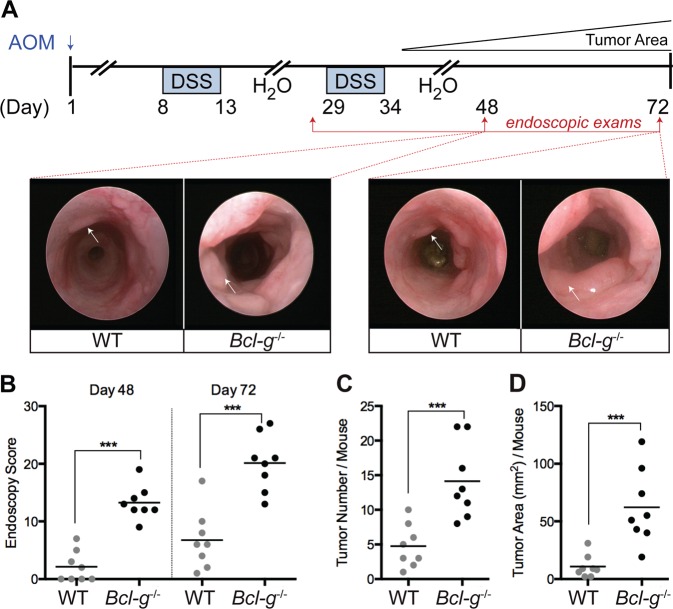
Loss of Bcl-G accelerates colitis-associated cancer. **a** Schematic representation of the colitis-associated cancer model with representative colonoscopy images of WT and *Bcl-g*^−/−^ mice at the indicated stage of the model. Images at day 48 and day 72 are shown for the same representative mouse of each genotype. **b** Colonoscopy scores for individual WT and *Bcl-g*^−/−^ mice at day 48 and day 72 of the model. *N* = 8 mice per genotype, ***P < 0.001. Student’s *t*-test. Representative of *N* > 3 experiments. **c** Total colonic tumor number for individual WT and *Bcl-g*^−/−^ mice on autopsy. *N* = 8 mice per genotype, ***P < 0.001. Student’s *t*-test. Representative of *N* > 3 experiments. **d** Total colonic tumor area for individual WT and *Bcl-g*^−/−^ mice on autopsy. *N* = 8 mice per genotype, ***P < 0.001. Student’s *t*-test. Representative of *N* > 3 experiments

We aged *Bcl-g*^−/−^ mice beyond one year and collected the entire GI tract to monitor for spontaneous tumor formation; however, no sporadic tumors were observed (data not shown). We next tested the hypothesis that loss of Bcl-G may predispose mice to tumor formation by subjecting *Bcl-g*^−/−^ and WT mice to the CAC model (Fig. 2a), whereby a single injection of the alkylating mutagen azoxymethane (AOM) leads to the sporadic induction of missense mutations in the *Ctnb1* gene in colonic epithelial cells, resulting in stabilization of β-catenin and aberrant activation of the Wnt signaling pathway [14, 15]. In this model, repetitive oral administration of the luminal irritant dextran sulfate sodium (DSS), which induces ‘flares’ of acute inflammation that mimic the mucosal damage observed in IBD patients, triggers the development of colonic tumors [16]. Monitoring of CAC progression by endoscopy showed that tumors appeared earlier and grew larger in *Bcl-g*^−/−^ mice than in their WT littermates (Fig. 2b). This was further apparent on autopsy, where overall colonic tumor number and burden (Fig. 2c, d) were significantly increased in *Bcl-g*^−/−^ mice. This observation suggests a potential tumor suppressive function for Bcl-G in the colon.

### Loss of Bcl-G does not alter sporadic gastrointestinal tumor onset or progression

**Fig. 3:**
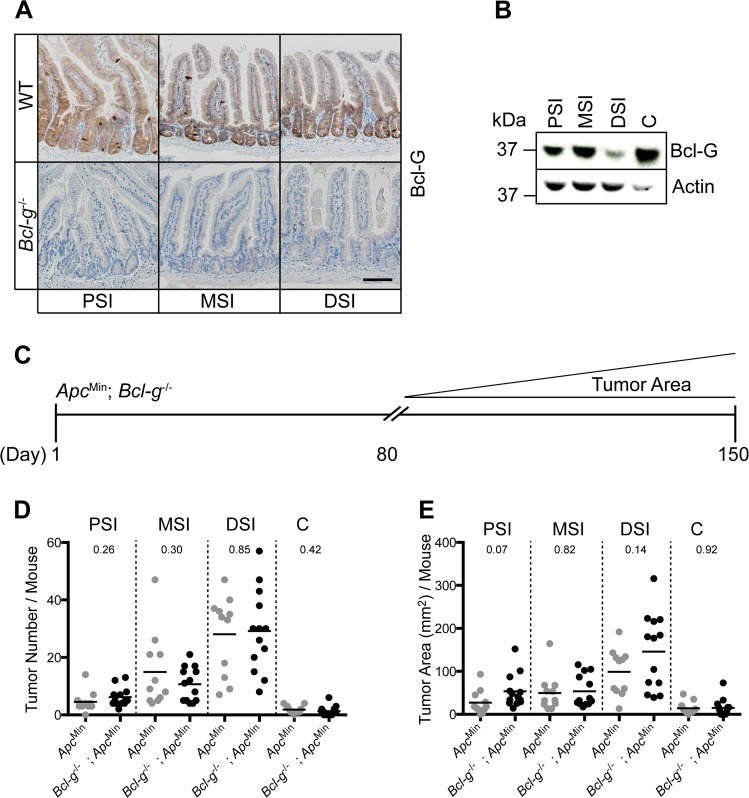
Loss of Bcl-g has no impact on the progression of sporadic gastrointestinal tumors. **a** Representative immunohistochemistry for Bcl-G in the proximal (PSI), middle (MSI), and distal (DSI) small intestine of 8-week-old WT and *Bcl-g*^-/-^ mice. Scale bar: 50 µm. **b** Representative immunoblot analysis of colonic epithelial cells isolated from the PSI, MSI, DSI, and colon of an 8-week-old WT mouse. Representative of *N* = 3 mice. **c** Schematic representation of tumor progression in the *Apc*^Min^ mouse model. **d** Total PSI, MSI, DSI, and colon **c** tumor number for individual age-matched (~120 days) WT and *Bcl-g*^−/−^ mice on autopsy. *N* > 11 mice per genotype. *P*-values are shown. **e** Total PSI, MSI, DSI, and C tumor area for individual age-matched (~120 days) WT and *Bcl-g*^−/−^ mice on autopsy. *N* > 11 mice per genotype. *P*-values are shown

Since Bcl-G is also highly expressed by epithelial cells of the small intestine in adult mice (Fig. 3a), we crossed *Bcl-g*^−/−^ mice to *Apc*^Min/+^ mice, which carry a germline truncation in *Apc* resulting in the development of small intestinal tumors following the spontaneous loss of heterozygosity of the WT *Apc* allele [17]. *Apc*^Min/+^ mice replicate the spontaneous formation of intestinal tumors found in familial adenomatous polyposis (FAP) patients [18]. Interestingly, we observed the lowest expression of Bcl-G in epithelial cells isolated from the distal small intestine (DSI) of naïve WT mice (Fig. 3b), which is where the greatest tumor burden in *Apc*^Min/+^ mice occurs. However, we did not observe a change in crypt morphology, crypt-villus length, or proliferation in the DSI of *Bcl-g*^−/−^ mice when compared to WT mice (Supplemental Fig. 2a-d), suggesting that loss of Bcl-G does not alter normal GI homeostasis in the DSI. We aged *Bcl-g*^−/−^;*Apc*^Min/+^ compound mutant mice and littermate *Apc*^Min/+^ mice to ~120 days, a time point when tumors are macroscopically detectable in *Apc*^Min/+^ mice (Fig. 3c). However, we observed no significant difference in the overall number (Fig. 3d) or size of the tumors (Fig. 3e) that were visible in the proximal, middle, or distal small intestine between the genotypes. We also observed no difference in the formation of sporadic tumors in the colon of *Bcl-g*^−/−^;*Apc*^Min/+^ mice compared to *Apc*^Min/+^ mice (Fig. 3d, e). Taken together, these observations suggest that the loss of Bcl-G has no impact on sporadic tumor onset or progression in the small intestine or colon.

### Bcl-G does not modulate tumor epithelial proliferation or apoptosis

In order to begin to understand how Bcl-G may contribute to CAC formation, we compared two of the most well recognized hallmarks of cancer: proliferation and evasion of apoptosis. We found that proliferation, indicated by Ki67-positive cells, was comparable between the tumors of WT and *Bcl-g*^−/−^ mice that had undergone the CAC model, and in the *Apc*^Min/+^ compared to *Bcl-g*^−/−^;*Apc*^Min/+^ mice (Supplemental Fig. 3a-d). We also observed no significant difference in the number of cells undergoing apoptosis, indicated by cleaved caspase-3-positive staining, in the tumors of *Bcl-g*^−/−^ mice compared to WT mice in either model (Supplemental Fig. 3e-h). Taken together, these observations suggest that Bcl-G does not contribute to the growth rate of tumors irrespective of whether or not they occur in a chronically inflamed environment.

The difference in tumor progression between the two models was not simply a reflection of the inflammation that was induced in the CAC model, since the level of tumor-infiltrating CD45-positive leukocytes was similar between tumors in each of WT and *Bcl-g*^−/−^ mice (Supplemental Fig. 4a-d). While there was a significant reduction in CD45+ leukocytes in the *Bcl-g*^−/−^;*Apc*^Min/+^ compared to *Apc*^Min/+^ mice (Supplemental Fig. 4d), this had no impact on the tumor burden. In the acute DSS model, there were no significant changes in weight-loss, colon length or histology (Supplemental Fig. 4e-g). Moreover, FACS characterization of the colonic lamina propria following acute DSS challenge revealed no changes in the influx of T cells or macrophages in *Bcl-g*^−/−^ mice compared to WT mice (Supplemental Fig. 4j–i). The lack of changes in mucosal inflammation suggests that an epithelial cell-intrinsic change triggered by mucosal damage contributes to the difference in *Bcl-g*^−/−^ mouse tumor burden in the CAC model.

### BCL-G expression is decreased in human GI pathologies

**Fig. 4:**
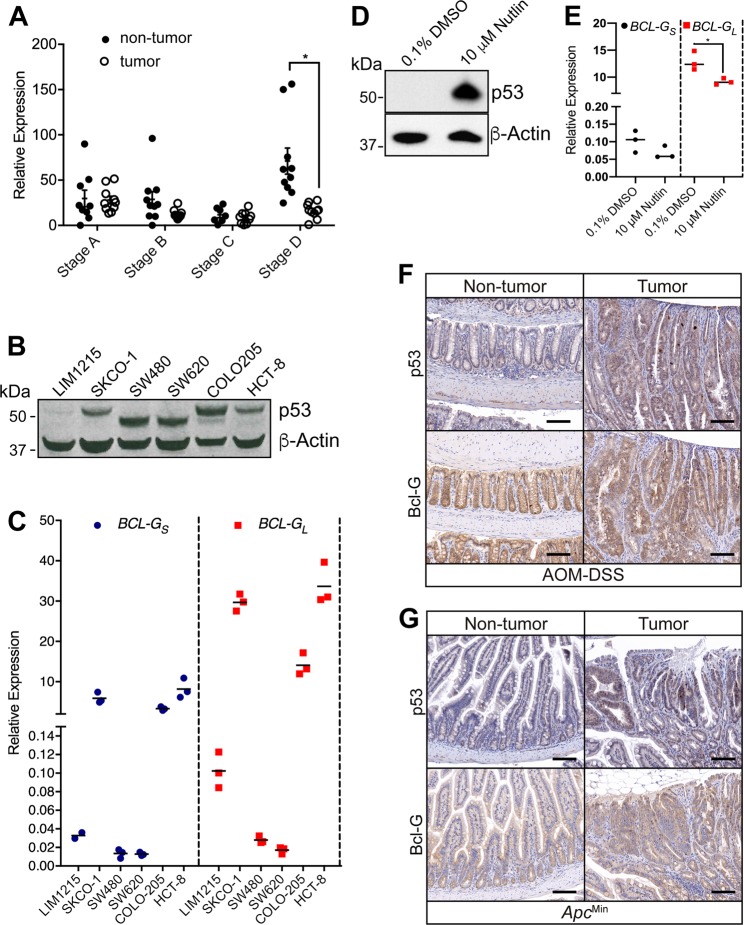
p53 expression does not correlate with Bcl-G expression in murine colonic tumors. **a** *BCL-G*_*L*_ mRNA expression from the non-tumor and adjacent tumor tissue collected from colorectal cancer patients with different stages of tumor progression. *P < 0.05. Student’s *t*-test. Data are presented relative to GAPDH as mean ± SEM. **b** Immunoblot for P53 in the indicated human colorectal cancer cell lines. **c** *BCL-G*_*s*_ and *BCL-G*_*L*_ mRNA expression in the colorectal cancer cell lines presented in **b**. Data for technical triplicates is presented relative to *GAPDH*. **d** Representative P53 immunoblot of the normal human colon cell line T4056 after treatment with 10 µM Nutlin-3 for 72 h. Representative of technical triplicates from two independent experiments. **e** *BCL-G*_*s*_ and *BCL-G*_*L*_ mRNA expression in the normal human colon cell line T4056 presented in **d**. Data for technical triplicates are presented relative to *GAPDH*. Representative of two independent experiments. *P < 0.05. Student’s *t*-test. **f** Representative p53 staining and Bcl-G staining in WT CAC tumors. Scale bar: 100 µm. **g** Representative p53 staining (arrows) and Bcl-G staining in *Apc*^Min^ DSI tumors. Scale bar: 100 µm

We explored the expression of *BCL-G*_*L*_ in human GI pathologies and observed a reduction in *BCL-G* expression in our own CRC patient cohort, which was specific to late stage tumors (Fig. 4a). We also observed a reduction in *BCL-G* expression in two publicly available colon cancer data sets (Supplemental Fig. 5a, b). Similarly, we observed a reduction in *BCL-G* expression in two publicly available data sets for human ulcerative colitis patients [19] (Supplemental Fig. 5c, d). To address the possibility that this reduction in *BCL-G*_*L*_ expression was attributed to changes in the proportion of epithelial cells in the inflamed patient tissue, we also examined the expression level of *EPCAM*, which is expressed exclusively by epithelial cells and found a comparable epithelial content between control and inflamed patients for one dataset (Supplemental Fig. 5e, f). These observations highlight that loss of BCL-G expression is a feature of human GI pathologies.

### Bcl-G expression is independent of p53 expression in colitis-associated cancer

p53 expression is a major player in the step-wise genetic sequence associated with colon cancers [20, 21]. In humans, mutations in TP53 that result in loss-of-function are an early event in chronic colitis and dysplasia [22–24]. In contrast, loss of TP53 expression occurs at the advanced stages of sporadic CRC [25]. TP53 has previously been shown to regulate *BCL-G* expression [26], in order to explore this relationship, we screened a panel of six human colon cancer cell lines, with different TP53 profiles (Fig. 4b) for *BCLG* expression. We observed that in cell lines with mutant TP53 (SW480 and SW620) the expression of *BCLG* was low, while in cell lines with wild-type TP53 (SKCO-1 and HCT-8) the expression of *BCLG* was high (Fig. 4c). An exception was LIM1215, which had low wild-type TP53 expression and low *BCLG* expression. COLO205 has controversial TP53 status, with both wild-type and mutations reported [27]. We observe a dominant wild-type TP53 band by western-blot, and elevated *BCLG* expression (Fig. 4b, c). In order to further explore a potential relationship between TP53 and *BCLG* expression, we stimulated the normal human colon cell line T4056 with 10μM of Nutlin-3 for 72 h to induce TP53 expression (Fig. 4d). Compared to the vehicle control we observed no changes in BCLG_S_ expression following treatment with Nutlin-3, while BCLG_L_ was mildly decreased (Fig. 4e). In the *Bcl-g*^−/−^ mice, which mimic loss of BCLG_L_, we detected no relationship between the expression of p53 and Bcl-G in the tumors of either of our colon cancer models (Fig. 4f, g).

### Bcl-G is expressed by LGR5+ colonic stem cells

**Fig. 5:**
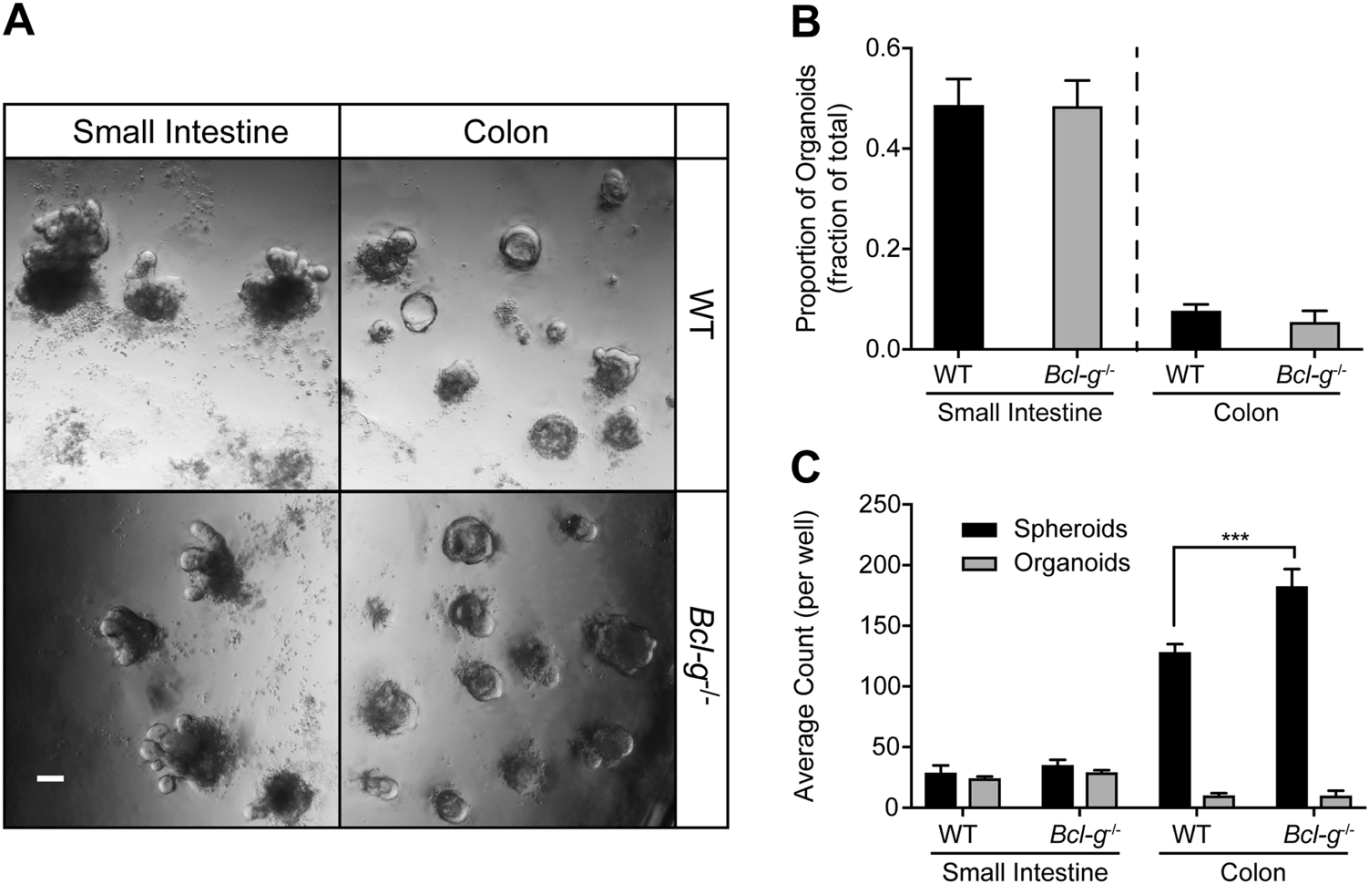
Loss of BclG does not alter regenerative potential of colonic epithelial cells. **a** Representative images of small intestine (day 7) and colonic (day 5) organoids generated from WT and *Bcl-g*^−/−^ mice. Scale bar: 100 µm. **b**, **c** Quantification of the proportion of organoids formed from single cell suspension (**b**), and the number of spheroids and organoids formed (**c**) in the small intestine or colon of WT and *Bcl-g*^−/−^ mice. ***P < 0.001. Student’s *t*-test. Data are presented as mean ± SEM

Colon cancer arises from a highly proliferative epithelium that is continuously renewed under steady-state conditions. Following epithelial injury in the colon, the homeostatic renewal process is replaced by a regenerative response [28], with highly active regenerative responses known to contribute to the formation of CRC tumors [29]. Since DSS-associated injury induces a mucosal wound healing response, we next sought to functionally test if there was a potential role for Bcl-G in the regeneration response, which is initiated by Lgr5+ stem cell populations [30]. To this end, we first established that *Bcl-g* was expressed by Lgr5+ cells, following FACS isolation of GFP^+^ cells from the colon of Lgr5-EGFP-IRES-CreERT [2] mice [30] (Supplemental Fig. 6a, b). We next tested a role for Bcl-G in the self-renewal process by generating organoids from the colon and small intestine of littermate WT and *Bcl-g*^−/−^ mice (Fig. 5a). We observed no difference in the proportion of organoids that were derived from either genotype (Fig. 5b), consistent with our observation that there was no change in proliferation of epithelial cells within the GI tract (Supplemental Fig. 3). We observed a significant increase in the ability of *Bcl-g*^−/−^ cells to generate spheroids (Fig. 5c), also referred to as colonospheres, which can be composed of several types of colonic epithelial cells. In contrast, we did not observe a change in the number of organoids, defined as multilobulated structures with a pseudolumen. Organoids develop from a spheroid by forming one or more buds (multicellular protrusions), and are also referred to as a colonoid. The increased number of spheroids, without a corresponding increase in organoids, suggests changes in the behavior of epithelial cells.

**Fig. 6:**
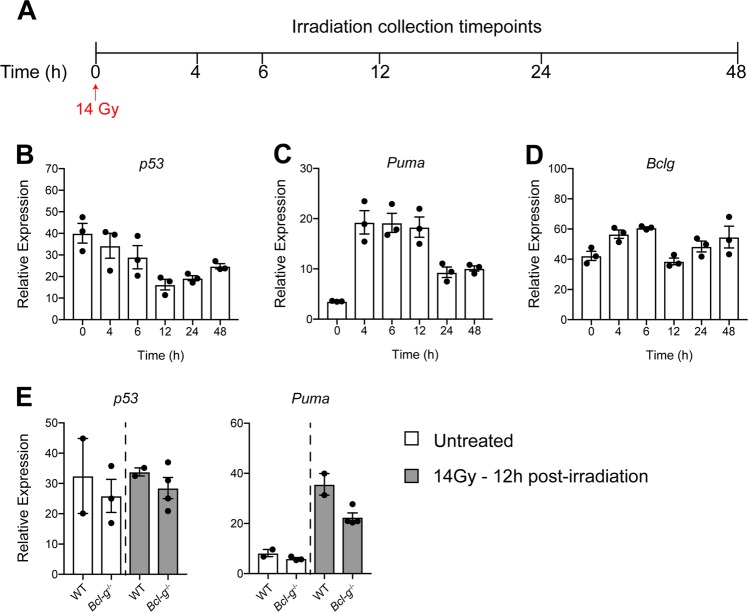
Loss of Bcl-G does not alter irradiation-induced damage in the intestine. **a** Schematic representation of the irradiation model used to study intestinal damage. Mice were lethally irradiated and collected at the indicated time points. **b**–**d** mRNA expression of *p53* (**b**), *Puma* (**c**), and *Bclg* (**d**) in WT mice at the indicated time points post-irradiation. *N* = 3 mice per time point. Data presented as mean ± SEM, relative to *Gapdh*. **e**, **f** mRNA expression of *p53* (**e**) and *Puma* (**f**) in WT and *Bcl-g*^−/−^ mice that were untreated and 12 h post-irradiation. *N* = 2–3 mice per time point. Data presented as mean ± SEM, relative to *Gapdh.*

To further explore the role of Bcl-G in a regenerative response, we lethally irradiated WT mice (Fig. 6a) and compared the expression of *p53*, *Puma* as an indicator of cell death, and *Bcl-g* in the epithelium of the small intestine as the most radio-sensitive compartment, which is routinely examined to assess the regenerative capacity following irradiation-induced epithelial cell death [31, 32]. Over the course of the regenerative response we found no correlation between any of the genes examined (Fig. 6b–d), further highlighting that *Bcl-g* expression is not associated with *p53*. On further comparison of irradiation-induced epithelial damage at 12 h, the peak of epithelial cell death, there was no visible histological difference between WT and *Bcl-g*^−/−^ mice (not shown) consistent with the lack of change in *p53* or *Puma* expression (Fig. 6e). Taken together, Bcl-G does not appear to contribute to epithelial cell survival.

### Loss of Bcl-G alters the colon mucin scaffolding network

**Fig. 7:**
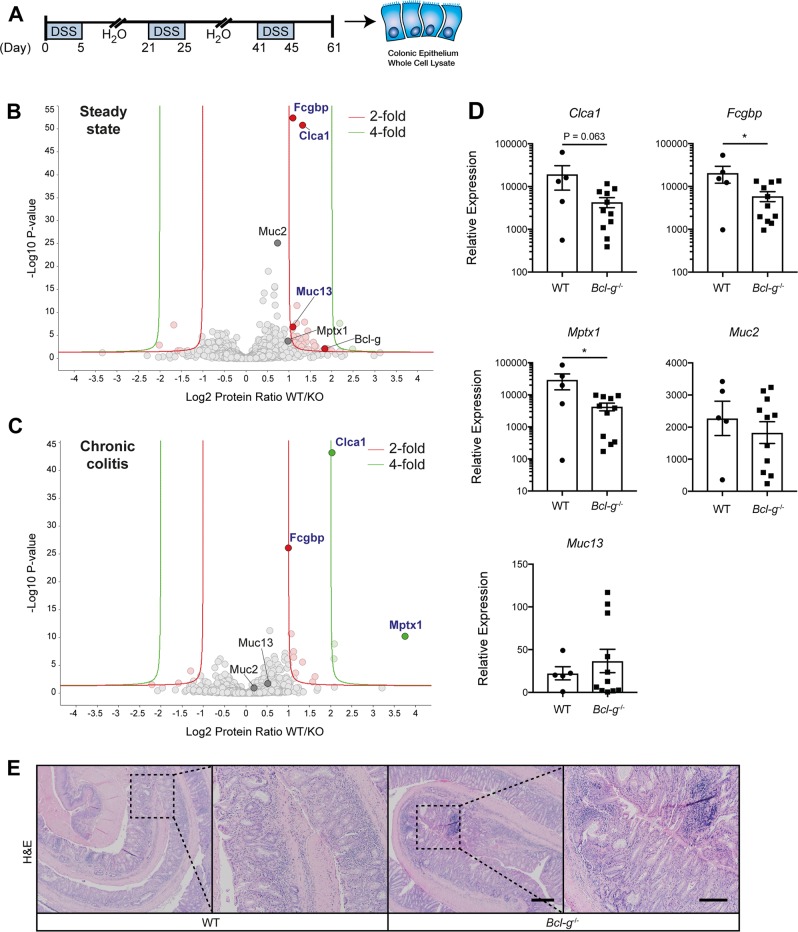
Loss of Bcl-g is associated with an altered mucin network. **a** Schematic representation of the chronic colitis model and workflow. **b** Volcano plot illustrating the log_2_ protein ratios in whole cell lysates of colonic epithelial cells isolated from naïve WT relative to littermate *Bcl-g*^−/−^ mice following quantitative MS analysis. Proteins were deemed differentially regulated if the log_2_ fold change in protein expression was greater than 2-fold (red) or 4-fold (green) and a –log_10_ *p*-value (with Benjamini-Hochberg correction) ≥ 1.3, equivalent to a *p*-value ≤ 0.05. **c** Volcano plot illustrating the log_2_ protein ratios in whole cell lysates from colonic epithelial cells isolated from chronic DSS-treated WT relative to littermate *Bcl-g*^−/−^ mice following quantitative MS analysis. Proteins were deemed differentially regulated in the log_2_ fold change in protein expression was greater than 2-fold (red) or 4-fold (green) and a –log_10_ *p*-value (with Benjamini-Hochberg correction) ≥ 1.3, equivalent to a *p*-value ≤ 0.05. **d** mRNA expression of mucus-associated genes *Clca1, Fcgbp, Mptx1, Muc2*, and *Muc13* in WT and *Bcl-g*^−/−^ mice following chronic colitis. *N* ≥ 5 mice per genotype. *P < 0.05. Student’s *t*-test. Data presented as mean ± SEM, relative to *Gapdh*. **e** Representative H&E sections of the distal colon from mice of the indicated genotype on day 61 of the model. Scale bar: 200 µm, inset: 100 µm

In order to examine how the epithelial response to DSS may promote tumor formation in the absence of Bcl-G expression, we employed a model of chronic colitis that most closely recapitulates the distal colonic mucosal damage associated with ulcerative colitis [33]. Mice were provided with three cycles of 2% DSS in their drinking water to mimic the recurring flares of mucosal damage experienced by IBD patients (Fig. 7a). No appreciable change in daily weights, colitis scores or colon lengths were observed (Supplemental Fig. 7). On Day 61 of this model, colonic epithelial cells were isolated and whole cell lysates were prepared for quantitative mass spectrometry (MS) analysis to identify global changes in the proteome compared to naïve, unchallenged mice at steady-state. While we observed a number of changes between naïve WT and *Bcl-g*^*−/−*^ mice including Bcl-G (Fig. 7b), as expected, we were most intrigued by the changes observed in mucins, and mucin-like proteins. Mucins are heavily O-glycosylated cell surface proteins that are a first line of defense in protecting the epithelial cells that line the GI tract [34]. Muc1 is known to be the predominant cell surface mucin in the stomach, while Muc2, Muc4, Muc12, and Muc13 are the primary cell surface mucins found in the intestine [35]. We detected reduced levels of Muc13 (1.10 Log_2_ fold) in naive *Bcl-g*^−/−^ mice compared with WT mice (Fig. 7b), although this was not observed at the gene level (Fig. 7d).

Fc-gamma binding protein (Fcgbp) is covalently attached to Muc2 to provide structural integrity to the mucus network, with recent proteomic analyses demonstrating that both proteins are expressed in the mucus granule of goblet cells found in the human colon [36]. We identified a significant reduction in the expression of Fcgbp in the *Bcl-g*^−/−^ mice compared with WT mice in both naïve mice (1.09 Log_2_ fold) and following DSS treatment (1.01 Log_2_ fold; Fig. 7b, c). We confirmed this observation at the expression level of the corresponding genes in DSS-treated epithelial cells (Fig. 7d). Chloride accessory channel 1 (Clca1), which has a similar expression pattern to Muc2 [37], was also significantly decreased in naïve *Bcl-g*^−/−^ mice (1.32 Log_2_ fold) and following DSS treatment (2.04 Log_2_ fold; Fig. 7b, c). In addition, we identified a significant reduction in the levels of Mucosal Pentraxin 1 (Mptx1) in *Bcl-g*^−/−^ mice following chronic DSS-induced mucosal damage (Fig. 7c), which was also reduced at the gene level (Fig. 7d). Mptx1 is a colon-specific marker for mucosal cell integrity that is highly expressed in a naïve colon, and strongly reduced upon epithelial damage [38], suggesting increased epithelial damage in *Bcl-g*^−/−^ mice, which was reflected by histopathology in the *Bcl-g*^−/−^ mice (Fig. 7e).

Collectively these data suggest that there are several changes that occur in the mucus scaffold within naïve *Bcl-g*^−/−^ mice that are also present following chronic DSS treatment, consistent with previous reports implicating changes to mucins in CRC.

## Discussion

Bcl-G is a poorly characterized member of the Bcl-2 family of proteins. The alternative splice acceptor site that is used to produce BCL-G_s_ is not conserved across other species, for this reason we utilized mouse Bcl-G, as the closest homolog to understand the biological function of human BCL-G_L_. We have demonstrated that mouse Bcl-G is highly expressed in the epithelial cells lining the crypt-villus structures of the small intestine and colon throughout development. The lack of a compensatory increase in other classic Bcl-2 family members in the adult colon when Bcl-G was genetically deleted suggests that the function of Bcl-G is unlikely to be shared by the other family members. This is consistent with the non-redundant roles for the various Bcl-2 family members in numerous other disease models [39–42].

The only overt functional phenotype in the colon reported for a Bcl-2 family member has been for Puma and Bcl-x_L_, with the genetic loss of Puma associated with reduced cell death and resistance to the clinical symptoms of DSS-induced colitis [43]. *Puma*^−/−^ mice have an increased tumor burden in the CAC model, and when crossed into the *Apc*^Min^ background [44]. Specific ablation of Bcl-x_L_ in the intestinal epithelium does not result in an overt phenotype; however, tumor burden is significantly reduced in the CAC model and associated with increased cell death [45]. By contrast, and consistent with our observations for Bcl-G, deficiency in Bad, Bim, Bax, Bak, or Noxa had no effect on epithelial apoptosis following acute DSS-induced colitis [43]. Bid, on the other hand, has been shown to have a non-apoptotic function during intestinal inflammation, with selective ablation of Bid in intestinal epithelial cells or macrophages resulting in a dampening of cytokine secretion and chemically induced colitis [46]. We have, however, demonstrated a unique sensitivity of *Bcl-g*^−/−^ mice to the CAC model.

The balance between homeostasis and the support of cancer progression is not well explored in vivo. Much of our knowledge is centered around the tumor suppressor gene p53, as a central factor for cell fate decisions and prevention of the expansion of damaged and mutated cells [47]. The activation of p53 in response to DNA damage or cellular stress leads to cell cycle arrest, apoptosis or senescence depending on the cellular context [48], with the loss of p53 in colonic epithelial cells known to increase their survival by reinstating protective factors that control inflammation leading to an increase in CAC incidence and dysplasia [49–51]. Previous studies have linked BCL-G and p53 expression, with suggestions that RNAi knockdown of *BCL-G* resulted in a significant reduction in p53 induced apoptosis, but not a complete ablation [48]. However, we did not observe a significant relationship between Bcl-G and p53 in the mouse colonic epithelium, in contrast to the observation that p53 can regulate *BCL-G* expression [26]. The discrepancies in our murine models may lie in the potential for different functions for BCL-Gs compared to BCL-G_L_, with previous publications not clearly indicating the BCL-G isoform examined.

Our quantitative proteomics analysis suggested that loss of Bcl-G results in the modulation of the mucin network, including Muc2 for example, which is the main gel-forming mucin secreted by goblet cells [52, 53], creating a physical barrier that protects the underlying epithelium. The question remains as to how Bcl-G regulates mucin levels. We do not attribute this observation to increased cell death in *Bcl-g*^−/−^ mice, resulting in lower levels of mucin producing cells, as we observed no changes in the length of the crypt-villus axis or the number of Caspase-3-positive cells at steady-state or in the cancer models. We believe that the contribution of Bcl-G to the mucin network is a reflection of a potential difference in the transition from proliferation to differentiation of colonic epithelial cells. While we observed no significant differences in Ki67+ proliferating cells in any of our models, loss of *Bcl-g* was linked to decreased *Clca1*, which is also decreased in CRC patients and is associated with inhibition of CRC epithelial differentiation when knocked down in cell lines [37, 54]. Similar to *Bcl-g*^−/−^ mice, *Clca1*^−/−^ mice display no overt phenotype in the acute DSS model, although mucous composition is not associated specifically with Clca1 expression [37, 54]. Alternatively, changes in the mucin network in *Bcl-g*^−/−^ mice may reflect changes in goblet cell restitution, a process that is not well understood.

The genetic loss of *Muc2* results in a depleted mucus layer and elevated levels of the pro-inflammatory cytokines TNF-α, IL-1β, and IFN-γ [55–57]. The expression of *BCL-G*_*L*_ has also been shown to be enhanced by both IFN-α and IFN-γ stimulation, through the ability of STAT1 and IRF-1 to interact with the BCL-G promoter [58]. We were also intrigued with the decrease in Muc13 expression that was observed in naïve *Bcl-g*^−/−^ mice, since increased expression of Muc13 has been detected in ovarian, gastric, and metastatic colon cancers [59–62]. In mouse models, Muc13 protects against intestinal epithelial damage and inflammation induced by DSS by protecting colonic epithelial cells from cell death through the activation of both intrinsic and extrinsic NF-κB signaling and subsequent upregulation of BCL-X_L_ [63]. Loss of Muc13 may represent a compensatory mechanism to maintain homeostasis in the absence of Bcl-G. In summary, we have demonstrated that the *Bcl-g*^−/−^ mice harbor a disrupted mucin network and thus are predisposed to colon tumorigenesis.

## Materials and methods

### Mice

All animal procedures were approved and conducted in accordance with guidelines established by the Walter and Eliza Hall Institute of Medical Research Animal Ethics Committee. Wild-type (WT) and littermate *Bcl-g*^-/-^ mice [9] on a C57BL/6 background were generated by heterozygote crosses (*Bcl-g*^+/−^), as were *Apc*^Min^ [17] and littermate *Apc*^Min^*:Bcl-g*^−/−^ mice, and heterozygous *Lgr5*-EGFP-IRES-creERT2 mice [30]. All mice were maintained under specific pathogen-free conditions at the Walter and Eliza Hall Institute for Medical Research. Two hours prior to euthanasia, mice received an intraperitoneal (i.p.) injection of Bromodeoxyuridine (BrdU, 50 mg/kg; Sigma–Aldrich, RPN201).

### Induction of mucosal damage

Acute mucosal damage was induced in gender and age-matched littermate mice by providing 1.5% (w/v) DSS dextran sulfate sodium (DSS; 36–50 kDa, MP Biomedicals, 0216110) *ad libitum* in the drinking water for 5 days followed by 3 days of normal drinking water. Chronic mucosal damage was induced in littermate gender- and age-matched mice by providing 2% (w/v) dextran sulfate sodium *ad libitum* in the drinking water for 5 days, followed by 16 days of normal drinking water, a cycle that was repeated three times. Colitis-associated cancer (CAC) was induced by providing azoxymethane (AOM; 10 mg/kg; Sigma–Aldrich, A5486) by i.p. injection 7 days prior to the chronic mucosal damage model (1.5% w/v DSS). Routine colonoscopy was performed and tumor burden scores were generated using a standard scoring system [33].

### Irradiation

To study epithelial regeneration, gender- and age-matched mice were exposed to a single dose of 14 Gy gamma radiation from a cobalt-60 source. After ethical euthanasia, the small intestine was removed and epithelial cells isolated following digestion in 3 mM EDTA for 1 h at room temperature. Following gentle shaking, the detached epithelial cells were separated by centrifugation and the epithelial pellet was processed for subsequent analysis.

### Induction of TP53

Cell lines were cultured to confluency in six-well plates, and left either treated with 0.1% DMSO (vehicle) or Nutlin-3 (10 μM, Cayman Chemicals Cat#18585) for 72 h. Cells were harvested, and prepared for Immunoblot or Real-time PCR analysis.

### Human subjects

Data from two NCBI GEO [64] human ulcerative colitis studies were downloaded and analyzed by GraphPad Prism. The following studies were used: GSE9452 (five control, 13 non-inflamed, and eight inflamed ulcerative colitis samples) [19] and GSE65114 (12 normal and 16 ulcerative colitis samples). Normalized expression levels for BCL-G_L_ and EPCAM were examined and pairwise Student’s *t*-test conducted to determine significant difference between groups (*P* < 0.05).

Gene expression data from RNA microarray analysis of tumor and non-tumor tissues from colorectal cancer patients were obtained through the publicly available ‘Oncomine Gene Browser’ database (ThermoFisher Scientific).

For gene expression data generated on our CRC patient cohort, all human tissues were obtained with informed patient consent and analyzed in accordance with approvals from the ethics committee at the Walter and Eliza Hall Institute of Medical Research. Tumor and adjacent normal (non-cancerous) tissues were analyzed from the same patient.

### Histological analysis

The excised colon from each mouse was opened longitudinally, “swiss rolled”, and fixed for 24 h in 10% (w/v) buffered formalin at room temperature. All tissues were embedded in paraffin, and 5-μm thick sections were stained with Haematoxylin and Eosin (H&E). Unstained sections were deparaffinised in xylene and rehydrated in a graded series of ethanol (100–70% v/v) finishing in dH_2_O. Antigen retrieval was achieved by boiling slides in citrate buffer (ThermoFisher Scientific, AP-9003-500) or EDTA-Tris (1 mM EDTA, 10 mM Tris pH 9) for 15 min, followed by incubation in 3% (v/v) H_2_O_2_ to inhibit endogenous peroxidases. Sections were blocked in 5% (v/v) goat serum and incubated with primary antibodies against Bcl-G (clone 2E11, [9]), BrdU (BD Biosciences, BD555627), Ki67 (Cell Signaling, CST 9129), Caspase-3 (Cell Signaling, CST 9664), CD45 (BD Biosciences, BD553076), or p53 (Leica Biosystems, P53-CM5P-L) overnight at 4 °C in a humidified chamber. Sections were rinsed, then incubated in HRP-conjugated biotinylated secondary antibodies for 1 h at room temperature, after which HRP (Vector Laboratories) was detected using a DAB Peroxidase kit (Dako, K346811-2). All sections were counterstained with Haematoxylin. The extent of positive staining per mm [2] of tissue was quantified using ImageJ software.

### Immunoblotting

Colonic tissue was lysed for 30 min in RIPA buffer containing 1 mM vanadate, protease, and phosphatase inhibitors. Proteins were separated on 4–12% Tris-glycine gradient gels (ThermoFisher Scientific, NP0321BOX) and transferred to nitrocellulose. After blocking in Odyssey buffer (LI-COR Biosciences, 927–40000), membranes were incubated in primary antibodies directed against Bcl-G (clone 2E11), p53 (Leica Biosystems), or Actin (Sigma, A2066, A4700) overnight at 4 °C, followed by the appropriate secondary antibody for 1 h at room temperature (LI-COR Biosciences, 926–32221, 926–68072, 926–68076). Proteins were visualized using the Odyssey infrared imaging system (LI-COR Biosciences).

### Quantitative proteomics analysis

Littermate age- and gender-matched mice were subjected to the chronic mucosal damage model or left untreated and isolated epithelial cells were prepared for protein lysis. Briefly, epithelial cells were isolated following digestion in 3 mM EDTA for 1 h at room temperature followed by lysis in TX-100 buffer. Protein concentrations of lysates were determined by the BCA method (ThermoFisher Scientific, 23225) prior to performing global proteome analysis.

Whole cell lysates (200 μg) derived from each biological replicate were prepared for mass spectrometry analysis using the FASP digestion method as previously described [65], with the following modifications. Proteins were reduced with Tris-(2-carboxyethyl)phosphine (TCEP; 10 mM final concentration, ThermoFisher Scientific, 20490), digested with sequence-grade modified Trypsin Gold (Promega, V5280) (1 μg for WCLs) in 50 mM NH_4_HCO_3_ and incubated overnight at 37 °C. Peptides were then eluted with 50 mM NH_4_HCO_3_ in two 40 μL sequential washes and acidified in 1% formic acid (final concentration).

MS analysis was performed as described previously [66]. Briefly, acidified peptide mixtures (1 μl) were subjected to nano-LC MS/MS analysis on a nanoAcquity system (Waters), coupled to a Q-Exactive Classic mass spectrometer. Raw files consisting of high-resolution MS/MS spectra were processed with MaxQuant (version 1.5.8.3) for feature detection and protein identification using the Andromeda search engine [67]. Extracted peak lists were searched against the UniProtKB/Swiss-Prot *Mus musculus* database (October 2016) and a separate reverse decoy database to empirically assess the false discovery rate (FDR) using strict trypsin specificity allowing up to two missed cleavages. The minimum required peptide length was set to seven amino acids. Modifications: Carbamidomethylation of Cys was set as a fixed modification, while N-acetylation of proteins and oxidation of Met were set as variable modifications. The mass tolerance for precursor ions and fragment ions were 20 ppm and 0.5 Da, respectively. The “match between runs” option in MaxQuant was used to transfer identifications made between runs on the basis of matching precursors with high mass accuracy [68]. PSM and protein identifications were filtered using a target-decoy approach at a false discovery rate (FDR) of 1%. Statistically-relevant protein expression changes between the WT and *Bcl-g*^*−*/−^ samples within the WCLs were identified using a custom in-house designed pipeline as previously described [66].

### Real-time PCR analysis

Total RNA was extracted from snap frozen tissue using TRIzol (ThermoFisher Scientific, 15596026) or an RNeasy Kit (Qiagen, 74136) as per manufacturer instructions. Reverse transcription was performed using the High Capacity cDNA Reverse Transcription Kit (ThermoFisher Scientific, 4368813). Real-time PCR amplification was achieved using Taqman (Bioline, BIO-83020) and SYBR (Bioline, QT605-20) reagents. The Taqman probes used were: *Bcl-g* (Mm01261010_m1), *Bcl2* (Mm00477631_m1), *Mcl1* (Mm01257351_g1), *Bcl-xl* (Mm00437783_m1), *Puma* (Mm00519268_m1), *Bim* (Mm00437796_m1), *Bax* (Mm00432051_m1), *Bak1* (Mm00432045_m1), *Tp53* (Mm01731290_g1)*, Muc2* (Mm00458299_m1), *Muc13* (Mm00495397_m1), and *Gapdh* (Mm99999915_g1). The SYBR green primers used were: *Clca1* (F: 5’-AACAACGGCTATGAGGGCAT-3’, R: 5’-TGAGTCACCATGTCCTTTATGTGT-3’), *Fcgbp* (F: 5’-GGCCTGGGGTAATGGGAAAG-3’, R: 5’-GTCCACACCGTTCACCTTGA-3’), *Gapdh* (F: 5’-CAACTCACTCAAGATTGTCAGCAA-3’, R: 5’-TACTTGGCAGGTTTCTCCAGGC-3’), *Mptx1* (5’-GAGGGTTGCTGATCTCCCA-3’, R: 5’-GCCTTTCCCTTCATGTCTGTTT-3’), *BCL-G* (short specific, F: 5’-CCAAAATTGTTGAGCTGCTG-3’, R: 5’-CATCAAACCATCCTGTGGAA-3’), *BCL-G* (long and short 1, F: 5’- AGGGTCTCTCCTTCCAGCTC-3’, R: 5’-TCTTTCCAACTGATCTCCTGAA-3’), *BCL-G* (long and short 2, F: 5’-CCAAAATTGTTGAGCTGCTG-3’, R: 5’-CAGAGTAGGACAGCCCATCC-3’) and *GAPDH* (F: 5’ GTCGGAGTCAACGGATT-3’. R: 5’-AAGCTTCCCGTTCTCAG-3’). All samples were run on a Viia7 Real-Time PCR System (ThermoFisher Scientific). Target gene expression is expressed relative to the housekeeping gene expression (*Gapdh*) is presented in arbitrary units (2^(-ΔCt)^).

### FACS analysis

The colon was opened longitudinally and washed with PBS to remove feces and cut into 5 mm pieces. The samples were then incubated in 10 mL pre-digestion mix (3 mM EDTA, 1 mM DTT, HBSS) for 20 min on ice. Mechanical release of epithelial cells was achieved by shaking for 30 s, after which the supernatant was passed through a 100-μm cell strainer into a new 50 mL tube and placed on ice. This was repeated twice with 5 mL pre-digestion mix, with pooling of the supernatant from previous steps. The tissue was then incubated in digestion mix (0.6 mg/mL collagenase/dispase, Sigma–Aldrich, HBSS) for 15 min at 37 °C. The samples were then shaken for 30 s, and then supernatant was passed through a 100-μm cell strainer and pooled with previous fractions. The cells collected from all flow through steps were then pelleted by centrifugation at 4 °C (5 min, 300 g), and the cell pellet was resuspended in 3 mL FACS buffer for processing for flow cytometry and cell sorting. PI was added at a final concentration of 1 μg/mL. Gating was performed to exclude doublet cells, PI-positive cells, and include LGR5-GFP-positive cells.

Lamina propria cells were isolated from colon by incubation in 1 mM EDTA/Ca^2+^Mg^2+^ free HBSS (ThermoFisher Scientific, 14170112) supplemented with 3% (v/v) FCS for 30 min at 37 °C with gentle shaking to remove epithelial cells. Colonic tissues were then minced and digested with 1 mg/ml of collagenase/dispase (Sigma–Aldrich, 11097113001) and 200μg/ml DNase I (Sigma–Aldrich, DN25-1G) in RPMI-1640 medium (ThermoFisher Scientific, 11875-093) supplemented with 3% (v/v) FCS for 45 mins at 37 °C with gentle shaking. Digested cells were filtered through a 70-μm cell strainer and mononuclear cells were isolated on a 37%/80% Percoll gradient (Sigma–Aldrich, 17-0891-01). Leukocytes were recovered from the interface and washed twice with FACS buffer (1 mM EDTA, 3% (v/v) FCS, PBS) before staining for FACS analysis.

Colonic single cell suspensions were stained with Fixable Viability Dye (ThermoFisher Scientific, 65-0866-18) to identify live cells. For surface marker staining, cells were stained with antibodies against the following markers: CD45.2 (BD Biosciences, BD560693), CD3ε (ThermoFisher Scientific, 25-0031-81), CD4 (ThermoFisher Scientific, 17-0041-81), MHCII (BD Biosciences, BD563413), CD64 (BD Biosciences, BD558539), and CD11b (BD Biosciences, BD562317). Flow cytometry was performed with a LSRFortessa^TM^ cytometer (BD Biosciences) and FlowJo analysis software (FlowJo).

### Reagents used for 3D colon and SI crypt culture

DMEM-F12 with GlutaMAX (ThermoFisher Scientific, 10565018) supplemented with penicillin-streptomycin (ThermoFisher Scientific, 15140-122), 1× N2 (ThermoFisher Scientific, 17502), 1× B27 (ThermoFisher Scientific, 17504), and HEPES (10 mM), R-spondin-Fc Conditioned Medium (2%), partially purified Wnt3a conditioned medium (1% v/v) (harvested from Wnt3A-expressing L cell line, ATCC no. CRL-2647 and affinity column purified as previously described [69]), recombinant human Noggin (100 ng/mL, PeproTech, 120-10), recombinant mouse EGF (50 ng/mL, PeproTech, 315-09) and Rho-kinase inhibitor Y27632 (10 µM, Sigma–Aldrich, Y0503). R-spondin-Fc conditioned medium was harvested from 293 F cells (after 7 days) transiently transfected with a human R-spondin2 construct that has Fc-fusion protein fused to the C-terminus and cloned into pApex vector [70, 71]. Y27632 was only added for the first 4 days of colon cultures. SI cultures do not require Wnt3A.

### Isolation of murine colon and small intestinal crypts

Detailed procedures to isolate normal murine colon crypts have been described previously [72]. The colons from WT and *Bcl-g*^−/−^ mice were sterilized in 0.04% (w/v) sodium hypochlorite solution for 10 min at room temperature, washed with PBS and incubated with 5 ml of chelation buffer (3 mM EDTA, 0.05 mM DTT in PBS) in for 60 min at room temperature. The colons were transferred in 2 mL PBS and the crypts were released into the PBS by manual shaking for 10 s. The supernatant (PBS with crypts) was collected and the procedure repeated four times, each with 2 mL fresh PBS. The colon was then discarded and the supernatant pelleted at 1000 rpm for 1 min at 4 °C. The steps were similar for the small intestines  (SI) except that they were incubated in chelation buffer for 5 min at 37 °C. The (SI) were then transferred into PBS and the villi were released by manual shaking 20 times, and the supernatant discarded. The SI fragments were then incubated in fresh chelation buffer for 20 min at room temperature. After which, the SI were transferred into PBS and manually shaken 20 times to release the SI crypts. This process was repeated three times, with the first round of supernatant discarded. The second and the third rounds of supernatants were pooled and pelleted down at 1000 rpm for 1 min at 4 °C.

### Crypt Culture setup

The required wells of a 96-well plate (BD Falcon) were coated with 15 µL of BD Matrigel™ matrix (BD Bioscience, 356237). The Matrigel was allowed to polymerize by incubating for 30 min at 37 °C. Crypt fragment suspension (containing 200 crypts in 100 µL volume) was added per well and incubated at 37 °C. The medium was replaced every other day.

### Crypt culture Microscopy and Image Processing

Detailed image acquisition and analysis procedures for acquiring images in 96-well plates were previously described [73]. Bright field microscopy was conducted using a retrofitted Nikon Eclipse Ti-U microscope using a ×4 objective lens and a motorized stage (Prior Scientific, H117). Bright field image stacks for each individual well were captured using a Nikon DS-Ri2 camera (Nikon Instruments Inc., MQA17000) driven by the NIS-Elements software (Nikon). The depth images of each FOV (well) were then stacked using the EDF algorithm in FIJI. Images were taken on most days for 9 days. The processed images were curated manually with the objects identified labelled either as S (spheroids) or O (organoids). The parameters were measured by drawing a line around the object. The average counts per well and the proportion of organoids were calculated.

### Statistical analysis

All data is presented as mean ± SEM and are representative of three or more experiments. Statistical significance was determined using a two-tailed Student’s *t*-test or ANOVA (parametric or non-parametric) with Bonferroni’s post-hoc as appropriate.

## Supplementary information

Revised Supplemental Information
