# Supplementary material for: Loss of Bcl-G, a Bcl-2 family member, augments the development of inflammation-associated colorectal cancer
Source: Cell Death Differ. 2019 Jul 11;27(2):742–57. doi: 10.1038/s41418-019-0383-9 (PMC7206067; doi:10.1038/s41418-019-0383-9)
Supplement: Supplementary file 1 — Revised Supplemental Information [file 41418_2019_383_MOESM1_ESM.docx]

**SUPPLEMENTAL INFORMATION**

**Loss of Bcl-G­­­, a Bcl-2 family member, augments the development of inflammation-associated colorectal cancer**

Paul M. Nguyen, Laura F. Dagley, Adele Preaudet, Nga Lam, Maybelline Giam, Ka Yee Fung, Kaheina Aizel, Gemma van Duijneveldt, Chin Wee Tan, Yumiko Hirokawa, Hon Yan K. Yip, Christopher G. Love, Ashleigh Poh, Akshay D’Cruz, Charlotte Burstroem, Rebecca Feltham, Suad Abdirahman, Kristy Meiselbach, Ronnie R.J. Low, Michelle Palmieri, Matthias Ernst, Andrew I. Webb, Tony Burgess, Oliver M. Sieber, Philippe Bouillet, Tracy L. Putoczki

**Supplemental Methods**

Supplemental Table 1. Table of key reagents used.

**Supplemental Figures**

Supplemental Figure 1, relates to Figure 1

Supplemental Figure 2, relates to Figure 3

Supplemental Figure 3, relates to Figure 2 and Figure 3

Supplemental Figure 4, relates to Figure 2 and Figure 3

Supplemental Figure 5, relates to Figure 4

Supplemental Figure 6, relates to Figure 5

Supplemental Figure 7, relates to Figure 7

Supplemental Table 2, relates to Figure 7

Supplemental Table 3, relates to Figure 7

**Supplemental Methods**

*Supplemental Table 1* **Table of key reagents used.**

| **REAGENT or RESOURCE** | **SOURCE** | **IDENTIFIER** |
| --- | --- | --- |
| **Antibodies** | | |
| Rat anti-mouse Bcl-G (clone 2E11) | Ref. 1 | N/A |
| Rabbit anti-mouse P53 (clone CM5) | Leica Biosystems | Cat#P53-CM5P-L |
| P53 (DO-1) | Santa Cruz | SC-126 |
| Rabbit anti-mouse Cleaved caspase 3 | Cell Signaling | Cat#CST 9664 |
| Rat anti-mouse CD45 | BD Biosciences | Cat#BD553076 |
| Rabbit anti-mouse Ki67 | Cell Signaling | Cat#CST 9129 |
| Rabbit or mouse anti-mouse Actin | Sigma | Cat#A2066, A4700 |
| Mouse anti-mouse BrdU | BD Biosciences | Cat#BD555627 |
| Mouse anti-mouse CD45.2 (clone 104, Alexa Fluor 700) | BD Biosciences | Cat#BD560693 |
| Rat anti-mouse MHCII (clone M5/114.15.2, BV605) | BD Biosciences | Cat#BD563413 |
| Mouse anti-mouse CD64 (clone X54-5/7.1, Alexa Fluor 647) | BD Biosciences | Cat#BD558539 |
| Rat anti-mouse CD11b (Clone M1/70, PE-CF594) | BD Biosciences | Cat#BD562317 |
| Rat anti-mouse CD4 (clone GK1.5, APC) | ThermoFisher Scientific | Cat#17-0041-81 |
| CD3ε (clone 145-2C11, PE-Cy7) | ThermoFisher Scientific | Cat#25-0031-81 |
| IRDye Goat anti-rabbit secondary antibody | LI-COR Biosciences | Cat#926-32221 |
| IRDye Goat anti-mouse secondary antibody | LI-COR Biosciences | Cat#926-68072 |
| IRDye Goat anti-rat secondary antibody | LI-COR Biosciences | Cat#926-68076 |
| **Bacterial and Virus Strains** | | |
|  |  |  |
| **Biological Samples** |  |  |
|  |  |  |
| **Chemicals, Peptides, and Recombinant Proteins** | | |
| Azoxymethane | Sigma-Aldrich | Cat#A5486 |
| Bromodeoxyuridine (BrdU) | Sigma-Aldrich | Cat#RPN201 |
| Collagenase/Dispase | Sigma-Aldrich | Cat#11097113001 |
| cOmplete Protease Inhibitor Cocktail | Sigma-Aldrich | Cat#11697498001 |
| Lab Vision citrate buffer (10x) | ThermoFisher Scientific | Cat#AP-9003-500 |
| Dextran Sulfate Sodium | MP Biomedicals | Cat#0216110 |
| Diaminobenzine (DAB) | Dako (Agilent) | Cat# K346811-2 |
| Fixable Viability Dye (eFluor 506) | ThermoFisher Scientific | Cat#65-0866-18 |
| Odyssey Blocking Buffer | LI-COR Biosciences | Cat#927-40000 |
| PhosSTOP | Sigma-Aldrich | Cat#04906845001 |
| Trizol | ThermoFisher Scientific | Cat#15596026 |
| Trypsin Gold | Promega | Cat#V5280 |
| Percoll | Sigma-Aldrich | Cat#17-0891-01 |
| DNase I | Sigma-Aldrich | Cat#DN25-1G |
| RPMI 1640 Medium | ThermoFisher Scientific | Cat#11875-093 |
| Hank’s Balanced Salt Solution (HBSS) | ThermoFisher Scientific | Cat#14170112 |
| DMEM-F12, GlutaMAX Supplement | ThermoFisher Scientific | Cat#10565018 |
| Penicillin-Streptomycin | ThermoFisher Scientific | Cat#15140-122 |
| N-2 Supplement | ThermoFisher Scientific | Cat#17502 |
| B-27 Supplement | ThermoFisher Scientific | Cat#17504 |
| Recombinant human Noggin | PeproTech | Cat#120-10 |
| Recombinant mouse EGF | PeproTech | Cat#315-09 |
| Y27632 | Sigma-Aldrich | Cat#Y0503 |
| Matrigel matrix | BD Bioscience | Cat#356237 |
| Iodoacetamide Bioultra | Sigma-Aldrich | Cat# I1149 |
| Tris-(2-carboxyethyl)phosphine (TCEP) | Pierce | Cat# 20490 |
| Ammonium Bicarbonate | Sigma-Aldrich | Cat#09830 |
| Formic acid | Merck | Cat#1.00264.2500 |
| **Critical Commercial Assays** | | |
| Avidin Biotin Complex ABC-kit with biotinylated secondary antibodies | Vector Laboratories | Cat#PK6101, 6102, 6104 |
| Bicinchoninic acid (BCA) Protein Assay Kit | ThermoFisher Scientific | Cat#23225 |
| High Capacity cDNA Reverse Transcription kit | ThermoFisher Scientific | Cat#4368813 |
| RNEasy Plus Mini Kit | QIAGEN | Cat#74136 |
| Sensimix II Probe No-rox | Bioline | Cat#BIO-83020 |
| SensiMix SYBR kit | Bioline | Cat#QT605-20 |
| 4-12% Bis-Tris NuPAGE gel system | ThermoFisher Scientific | Cat#NP0321BOX |
| **Deposited Data** | | |
|  |  |  |
| **Experimental Models: Cell Lines** | | |
| Mouse: Wnt3A-expressing L cell line | ATCC | CRL-2647 |
| Human: FreeStyle 293-F | ThermoFisher Scientific | Cat#R79007 |
| **Experimental Models: Organisms/Strains** | | |
| Wild-type C57BL/6 mice | WEHI | The Jackson Laboratory |
| *Bcl-g*^-/-^ | Ref. 2 |  |
| *Apc*^Min^ | Ref. 3 |  |
| *Lgr5*-EGFP-IRES-creERT2 | Ref. 4 |  |
| Oligonucleotides | | |
| Clca1 (F: AACAACGGCTATGAGGGCAT,  R: TGAGTCACCATGTCCTTTATGTGT) | This paper |  |
| Fcgbp (F: GGCCTGGGGTAATGGGAAAG,  R: GTCCACACCGTTCACCTTGA) | This paper |  |
| Gapdh (F: CAACTCACTCAAGATTGTCAGCAA,  R: TACTTGGCAGGTTTCTCCAGGC) | This paper |  |
| Mptx1 (F: GAGGGTTGCTGATCTCCCA,  R: GCCTTTCCCTTCATGTCTGTTT) | This paper |  |
| BCL-G (short specific, F:CCAAAATTGTTGAGCTGCTG, R: CATCAAACCATCCTGTGGAA) | This paper |  |
| BCL-G (Long and short 1,  F: AGGGTCTCTCCTTCCAGCTC,  R: TCTTTCCAACTGATCTCCTGAA) | This paper |  |
| BCL-G (Long and short 2,  F: CCAAAATTGTTGAGCTGCTG,  R: CAGAGTAGGACAGCCCATCC) | This paper |  |
| *Bak1* | ThermoFisher Scientific | Mm00432045_m1 |
| *Bax* | ThermoFisher Scientific | Mm00432051_m1 |
| *Bcl2* | ThermoFisher Scientific | Mm00477631_m1 |
| Bcl-G (*Bcl2l14*) | ThermoFisher Scientific | Mm01261010_m1 |
| Bcl-G (*BCL2L14*) | Thermo Fisher Scientific | Hs01030396_m1 |
| Bcl-x_L_ (*Bcl2l1*) | ThermoFisher Scientific | Mm00437783_m1 |
| Bim (*Bcl2l11*) | ThermoFisher Scientific | Mm00437796_m1 |
| *Gapdh* | ThermoFisher Scientific | Mm99999915_g1 |
| *GAPDH* | ThermoFisher Scientific | Hs99999905_m1 |
| *Mcl1* | ThermoFisher Scientific | Mm01257351_g1 |
| *Muc2* | ThermoFisher Scientific | Mm00458299_m1 |
| *Muc13* | ThermoFisher Scientific | Mm00495397_m1 |
| P53 (*Trp53*) | ThermoFisher Scientific | Mm01731290_g1 |
| Puma (*Bbc3*) | ThermoFisher Scientific | Mm00519268_m1 |
| **Recombinant DNA** | | |
| R-spondin2 construct | Ref. 5 |  |
| pApex vector | Ref. 6 |  |
| **Software and Algorithms** | | |
| CaseViewer (ver. 2.8) | 3DHISTECH | www.3dhistech.com/caseviewer |
| FIJI (ImageJ) | ImageJ | <https://fiji.sc/> |
| FlowJo (ver. 10.1r5) | FlowJo (BD) | www.flowjo.com |
| GraphPad Prism | GraphPad Prism | www.graphpad.com |
| MaxQuant (ver. 1.5.8.3) | Computation Systems Biochemistry (Max Planck Institute of Biochemistry | http://www.coxdocs.org/doku.php?id=maxquant:start |
| Oncomine | ThermoFisher Scientific | www.oncomine.org |
| Search Tool for the Retrieval of Interacting Genes (ver. 10.5) | STRING Consortium | https://string-db.org |
| NIS-Elements (Basic Research ver. 4.40) | Nikon Instruments Inc. | https://www.nikoninstruments.com/Products/Software/NIS-Elements-Basic-Research |
| **Other** | | |
| DynaMag-2 Magnet | ThermoFisher Scientific | Cat#12321D |
| Nikon Eclipse Ti-U microscope | Nikon |  |
| H117 – 114 x 75mm Travel Stepper Motor XY Stage | Prior Scientific | Cat#H117 |
| Nikon DS-Ri2 camera | Nikon Instruments Inc. | Cat# MQA17000 |
| Vivacon500 30k Hydrosart (FASP digestion) | Sartorius Stedim | Cat# VN01H22 |

**Supplemental Figures**

*
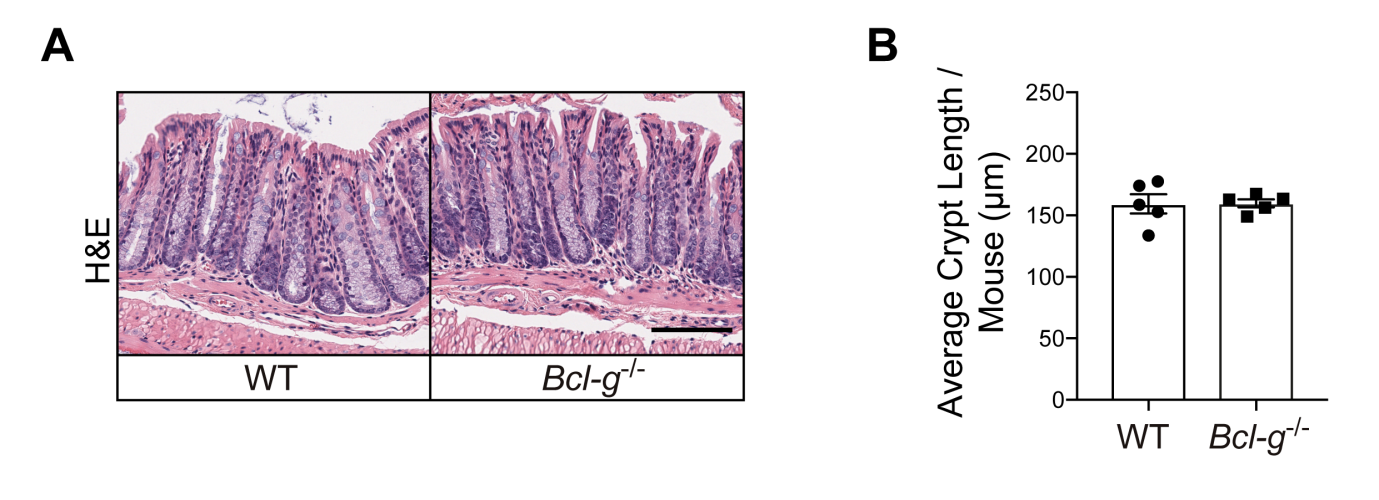
*

*Supplemental Figure 1.* ***Gastrointestinal homeostasis is normal in Bcl-g^-/-^ mice***

(A) Representative H&E sections from age- and gender-matched 8-week-old WT and *Bcl-g*^-/-^ mice. Scale bar: 100 μm.

(B) The colonic crypt length of individual age- and gender-matched 8-week-old WT and *Bcl-g*^-/-^ mice. Greater than 30 well oriented crypts per mouse were measured from the base of the crypt to the tip of the crypt using ImageJ software. N=5 mice per genotype. Data presented is mean ± SEM.

*
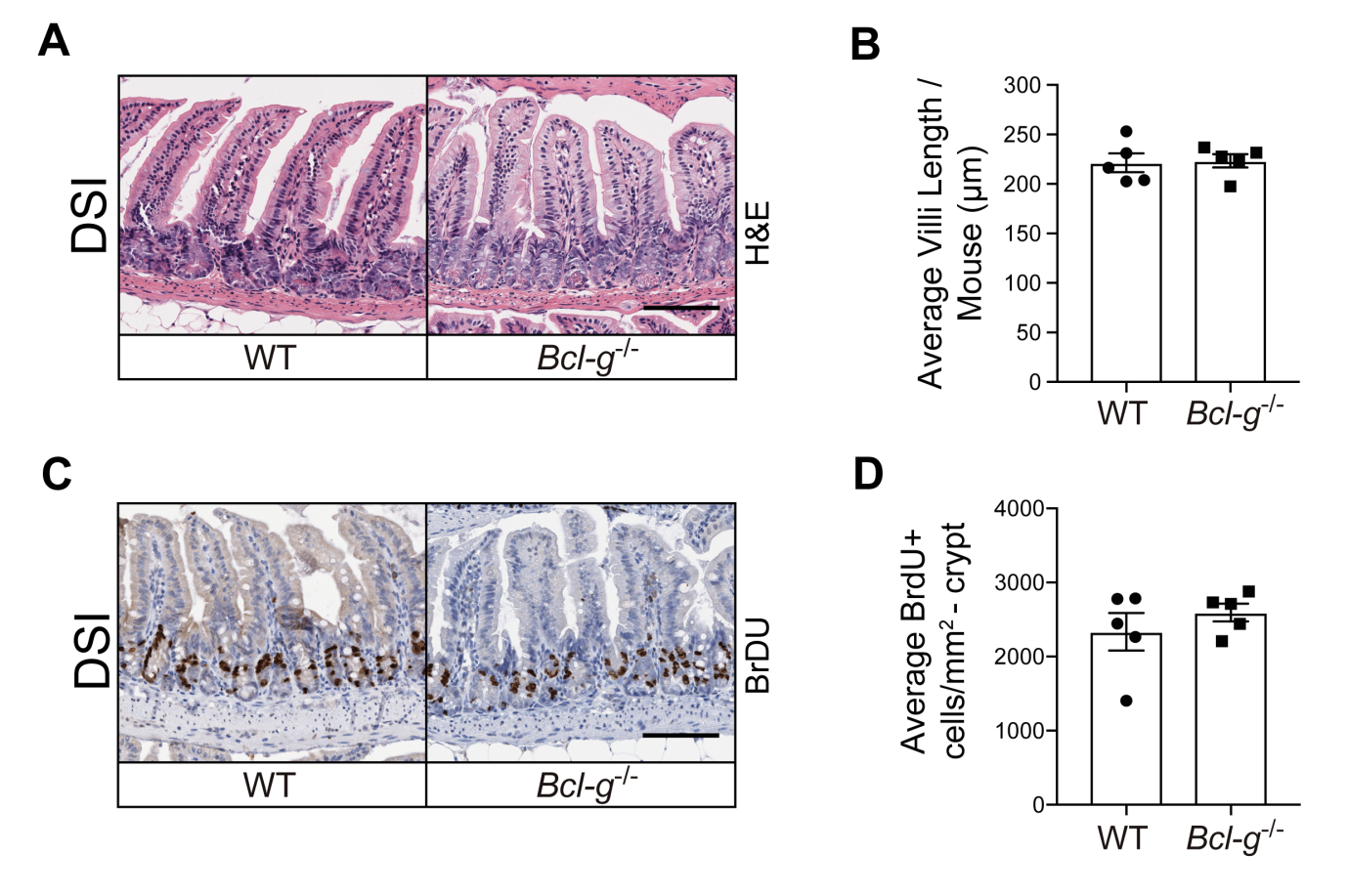
*

*Supplemental Figure 2* ***The distal small intestine is unaltered in Bcl-g^-/-^ mice***

(A) Representative H&E images of the distal small intestine (DSI) of age and gender matched 8-week-old WT and *Bcl-g*^-/-^ mice. Scale bar: 100µm.

(B) The DSI villi length of individual age and gender matched 8-week-old WT and *Bcl-g*^-/-^ mice. Greater than 30 well oriented villi per mouse were measured from the base of the crypt to the tip of the villi using ImageJ software. N=5 mice per genotype. Data presented is mean ± SEM.

(C) Representative immunohistochemistry for BrdU in the DSI of 8-week-old WT and *Bcl-g*^-/-^ mice. Scale bar: 100µm.

(D) Quantification of BrdU positive (+) staining in the DSI of individual WT and *Bcl-g*^-/-^ mice is shown. BrdU positive cells reside within the crypt structures. N=5 mice per genotype. Data presented is mean ± SEM.

*Supplemental Figure 3* **Loss of Bcl-G does not alter proliferation or apoptosis in colitis-associated and sporadic colorectal cancer models**

(A) Representative Ki67 staining of WT and *Bcl-g*^-/-^ colitis-associated tumors. Scale bar: 1mm, inset: 100µm.

(B) Quantification of Ki67 positive (+) cells in the colitis-associated tumors of individual mice. Each data point represents the average of all tumors within an individual mouse. Data presented is mean ± SEM.

(C) Representative Ki67 staining of WT and *Bcl-g*^-/-^ sporadic colon and DSI tumors. Scale bar: 100µm.

(D) Quantification of Ki67 positive (+) cells in sporadic colon and DSI tumors of individual mice. Each data point represents the average of all tumors within an individual mouse. Data presented is mean ± SEM.

(E) Representative Cleaved Caspase-3 staining (arrows) of WT and *Bcl-g*^-/-^ colitis-associated tumors. Scale bar: 1mm, inset: 100µm.

(F) Quantification of Cleaved Caspase-3 positive (+) cells in the colitis-associated tumors of individual mice. Each data point represents the average of all tumors within an individual mouse. Data presented is mean ± SEM.

(G) Representative Cleaved Caspase-3 staining of WT and *Bcl-g*^-/-^ sporadic colon and DSI tumors. Scale bar: 100µm.

(H) Quantification of Cleaved Caspase-3 positive (+) cells in the sporadic colon and DSI tumors of individual mice. Each data point represents the average of all tumors within an individual mouse. Data presented is mean ± SEM.

*Supplemental Figure 4* **Loss of Bcl-G does not alter inflammation in colitis-associated and sporadic colorectal cancer models**

(A) Representative CD45 staining (arrows) of WT and *Bcl-g*^-/-^ colitis-associated tumors. Scale bar: 1mm, inset: 100µm.

(B) Quantification of CD45 positive (+) cells in the colitis-associated tumors of individual mice. Each data point represents the average of all tumors within an individual mouse. Data presented is mean ± SEM.

(C) Representative CD45 staining of WT and *Bcl-g*^-/-^ sporadic DSI tumors. Scale bar: 100µm.

(D) Quantification of CD45 positive (+) cells in the sporadic DSI tumors of individual mice. Each data point represents the average of all tumors within an individual mouse. * P < 0.05. Student’s t-test. Data presented is mean ± SEM.

(E) Weight-loss (% of original) of mice of the indicated genotypes throughout the acute DSS mucosal damage model. N>5 mice per group. Representative of >3 independent experiments.

(F) Colon length of mice of the indicated genotypes from (E). N>5 mice per group. Representative of >3 independent experiments.

(G) Representative histology (H&E) of mice of the indicated genotype. Scale bar: 50 µm.

(H) Representative FACS plots from the colon lamina propria of WT and *Bcl-g*^-/-^ mice following an acute DSS-induced colitis model (1.5% DSS for 5 days, followed by 3 days of normal drinking water).

(I) Quantification of the data presented in (H). N=5 mice per genotype. Data is presented as mean ± SEM for individual mice of the indicated genotype.

*Supplemental Figure 5* **BCL-G expression in human CRC datasets.**

(A-B) Data obtained from Oncomine from the TGCA and Kaiser Colon study. One-way ANOVA (non-parametric) with Dunn’s multiple comparison test is presented.

(C) Relative *BCL-G_L_* mRNA expression in Control, and paired Uninflamed and Inflamed tissues from UC patients represented as box-whisker plots from microarray data set GSE9452.

(D) Relative *BCL-G_L_* mRNA expression in Normal and Colitis tissues from UC patients represented as box-whisker plots from microarray data set GSE65114.

(E) Relative *EPCAM* mRNA expression in Control, and paired Uninflamed and Inflamed tissues from UC patients represented as box-whisker plots from microarray data set GSE9452.

(F) Relative *EPCAM* mRNA expression in Normal and Colitis tissues from UC patients represented as box-whisker plots from microarray data set GSE65114.

*BCL-G_L_* mRNA expression in (C) and (E) were normalized using the Human Genome U133 Plus 2.0 Array normalization controls from Affymetrix. *P<0.05, **P<0.01, ***P<0.001, ****P<0.0001, ns: not significant.

*Supplemental Figure 6.* **Loss of Bcl-G expression does not alter regenerative potential**

(A) Representative FACS gating strategy for the isolation of GFP+ Lgr5+ cells from the colon of mice. (B) mRNA expression of *Bcl-g* in purified Lgr5 cell populations from the colon. Data is presented as mean ± SEM for 3 pooled mice per data point.

*Supplemental Figure 7.* **Loss of Bcl-G expression does not alter chronic colitis symptoms**

(A) Weight-loss (% of initial body weight) of mice of the indicated genotype throughout the chronic DSS protocol (outlined in Figure 7A). N>7 mice. Representative of >3 independent experiments.

(B) Colitis endoscopy scores during the second week of water in each DSS cycle. N>7 mice. Representative of >3 independent experiments.

(C) Colon length for the individual mice presented in (A-B). N>7 mice. Representative of >3 independent experiments.

*Supplemental Table 2* **Summary of proteins that are significantly altered in WCLs from mice that were either naïve or had undergone induction of chronic colitis.**

| **UniProt Accession** | **Gene name** | **Protein name** | **Log_2_ FC** | **-log_10_ P value** |
| --- | --- | --- | --- | --- |
| WT + DSS (WCL) | | | | |
| Q9D7Z6 | Clca1 | Calcium-activated chloride channel regulator 1 | 2.04 | 6.51E-44 |
| E9Q9C6 | Fcgbp | IgGFc-binding protein | 1.01 | 8.37E-27 |
| Q8R1M8 | Mptx1 | Mucosal pentraxin | 3.75 | 6.17E-11 |
| WT - DSS (WCL) | | | | |
| Q9D7Z6 | Clca1 | Calcium-activated chloride channel regulator 1 | 1.32 | 1.74E-51 |
| E9Q9C6 | Fcgbp | IgGFc-binding protein | 1.09 | 4.50E-53 |
| Q8R1M8 | Mptx1 | Mucosal pentraxin | 3.75 | 6.17E-11 |
| Q3V1S6 | Muc13 | Mucin-13 | 1.10 | 1.48E-07 |
| Q8R1T4 | Muc2 | Mucin-2 | 0.73 | 7.34E-26 |

*Supplemental Table 3*. **Summary of proteins with significantly dysregulated expression in WCLs of naïve or chronic DSS-treated mice (complete table).**

**References**

1. Giam M, Mintern JD, Rautureau GJ, Hinds MG, Strasser A, Bouillet P. Detection of Bcl-2 family member Bcl-G in mouse tissues using new monoclonal antibodies. *Cell Death Dis* 2012, **3:** e378.

2. Giam M, Okamoto T, Mintern JD, Strasser A, Bouillet P. Bcl-2 family member Bcl-G is not a proapoptotic protein. *Cell Death Dis* 2012, **3:** e404.

3. Moser AR, Pitot HC, Dove WF. A dominant mutation that predisposes to multiple intestinal neoplasia in the mouse. *Science* 1990, **247**(4940)**:** 322-324.

4. Barker N, van Es JH, Kuipers J, Kujala P, van den Born M, Cozijnsen M*, et al.* Identification of stem cells in small intestine and colon by marker gene Lgr5. *Nature* 2007, **449**(7165)**:** 1003-1007.

5. Yip HYK, Tan CW, Hirokawa Y, Burgess AW. Colon organoid formation and cryptogenesis are stimulated by growth factors secreted from myofibroblasts. *PLOS ONE* 2018, **13**(6)**:** e0199412.

6. Evans MJ, Hartman SL, Wolff DW, Rollins SA, Squinto SP. Rapid expression of an anti-human C5 chimeric Fab utilizing a vector that replicates in COS and 293 cells. *Journal of Immunological Methods* 1995, **184**(1)**:** 123-138.
